# Promoting responsive care and early learning practices among caregivers of children 0–23 months in the Kyrgyz Republic: findings from integrating a counselling intervention with nutrition services

**DOI:** 10.1017/S1368980024001642

**Published:** 2024-10-08

**Authors:** Lesley Oot, Veronica Varela, Cholpon Abdimitalipova, Marie Paul Nisingizwe, Kristen Cashin, Begimai Zhumgalbekova, Kelsey Torres, Malia Uyehara, Kathryn Beck, Tim Williams, Nazgul Abazbekova, Saikalbubu Bozova, Cholponai Umurzakova, Jennifer Yourkavitch, Romilla Karnati, Catherine Kirk

**Affiliations:** 1 USAID Advancing Nutrition, 2733 Crystal Drive, Fourth Floor, Arlington, VA 22201, USA; 2 JSI Research and Training Institute, 44 Farnsworth Street, Boston, MA 02210, USA; 3 USAID Advancing Nutrition Kyrgyz Republic, 15 Razzakov str., office #9, Bishkek 720040, Kyrgyz Republic; 4 University of British Columbia, School of Population and Public Health, Vancouver V6T2C5, Canada; 5 Save the Children US, 501 Kings Highway E, Fairfield, CT 06825, USA; 6 Results for Development, 1111 19th St NW, Washington, DC 20036, USA; 7 ZemiTek LLC, USAID’s Global Solution Ventures, 1300 Pennsylvania Avenue NW, Washington, DC 20004, USA

**Keywords:** Nurturing care, Early childhood development, Parenting, Responsive care, Early learning, Complementary feeding

## Abstract

**Objective:**

To assess changes in caregiver practices for young children after integrating the *Responsive Care and Early Learning (RCEL) Addendum* package into nutrition services after 10 months of implementation.

**Design:**

We measured changes in RCEL practices through a pre- and post-intervention assessment comprising a household survey and observations. To implement the intervention, we trained health service staff and community volunteers to deliver RCEL counselling to caregivers of children 0–23 months of age through existing community and facility-level platforms.

**Setting:**

Jalal-Abad and Batken regions in the Kyrgyz Republic.

**Participants:**

Caregivers of children aged 0–23 months at baseline.

**Results:**

We found statistically significant increases in RCEL practices, availability of early learning opportunities in the home, decreases in parenting stress and improvements in complementary feeding practices after the intervention implementation period.

**Conclusions:**

Findings show that delivery of RCEL counselling using the *RCEL Addendum* was associated with improved responsive care practices and early learning opportunities. We also found that integration of RCEL with infant and young child feeding counselling did not disrupt nutrition service delivery or negatively affect complementary feeding outcomes, but rather suggest synergistic benefits. Given the importance of providing holistic care to support optimal early childhood development, these findings provide new evidence on how to strengthen the delivery of nurturing care services in the Kyrgyz Republic.

Globally, around 250 million children in low- and middle-income countries (LMIC) under age 5 are at risk of not meeting their developmental potential^(1)^. To grow and develop optimally, children need nurturing care, which is composed of five interrelated components – safety and security, good health, responsive caregiving, adequate nutrition and early learning opportunities^(2)^. Nurturing care is particularly important during the first 3 years of life, when a child’s brain is growing most rapidly^(3)^. Global research shows that integrated approaches that provide holistic care, such as combining nutrition and responsive care and early learning (RCEL) interventions, result in better outcomes for children^(4–6)^. Therefore, the World Health Organization recommends integrating nutrition programming with RCEL activities^(6)^.

To support global recommendations for more integrated and holistic programming for young children, USAID Advancing Nutrition, the U.S. Agency for International Development’s (USAID) flagship multi-sectoral nutrition project, created a global resource – the *Responsive Care and Early Learning (RCEL) Addendum* ^(7)^. The *RCEL Addendum* is a package of materials developed to facilitate the training and support of community and facility-based counsellors who provide counselling on two components of nurturing care (RCEL). These components were missing from existing infant and young child feeding (IYCF) counselling packages, such as the *Community Infant and Young Child Feeding (C-IYCF) Counselling Package* ^(8)^. Developed between 2019 and 2021, and updated in 2023, the *RCEL Addendum* includes counselling cards; training materials and a planning, adaptation and implementation guide. The package focuses on the following topics: responsive care, responsive feeding, early learning, monitoring children’s development, caregiver well-being and supporting children with feeding difficulties^(8)^. We hypothesised that quality implementation of the *RCEL Addendum* would result in improved caregiver practices related to responsive caregiving and feeding, reductions in parental stress, improvements in opportunities within the home for early learning and have no negative impacts on IYCF behaviours. The purpose of this study was to assess changes in caregiver practices for young children (0–23 months of age) associated with integrating the RCEL Addendum into existing community- and facility-level nutrition and child health services in the Kyrgyz Republic by assessing changes in caregiver RCEL practices, following 10 months of a RCEL counselling intervention. The results of the study will inform specific opportunities for strengthening nurturing care to improve early childhood development (ECD), as well as specific ways to improve the *RCEL Addendum* package overall.

In the Kyrgyz Republic, a LMIC in Central Asia, access to health and nutrition services and primary education is very high—98 % of women give birth in a health facility and nearly 100 % of the adult population is literate^(9)^. The risk of poor development among children under age 5 due to stunting and extreme poverty declined dramatically from 33 % in 2005 to 14 % in 2015^(10)^. However, largely due to the war in Ukraine and the COVID-19 pandemic, childhood poverty and its associated negative outcomes on child development and nutrition are rising in Central Asia^(1,11)^. Additionally, 28 % of children ages 3–5 years are not developmentally on track, and only 24 % of children have access to childcare centres or preschools to support early learning^(10,12)^.

The Government of the Kyrgyz Republic called for investment in ECD in the National Development Strategy (2018–2040) and enacted a law in 2017 emphasising the importance of quality care and education for children aged 0–7 years^(13)^. However, a 2021 landscape analysis showed continued gaps in the policy environment (i.e. no multi-sectoral ECD strategy) to support optimal ECD outcomes^(14)^. Furthermore, with limited RCEL-focussed programming in the Kyrgyz Republic, parents often lack the knowledge and skills to effectively engage with their children^(14,15)^. Therefore, given the Government’s interest in improving ECD, the clear need for supportive services for caregivers and a USAID Advancing Nutrition project currently providing support for quality IYCF counselling, the Kyrgyz Republic was an ideal location to test the *RCEL Addendum*.

## Methods

### Study setting

The Kyrgyz Republic is a landlocked, mountainous country of approximately 7 million people. The study was conducted in seven rayons (districts) across two oblasts (regions) of the Kyrgyz Republic: Batken, Kadamjay and Leilek rayons in the Batken oblast and Aksy, Bazar-Korgon, Nooken and Suzak rayons in the Jalal-Abad oblast. The two oblasts have a combined population of 1 881 905, with an estimated 305 233 children under the age of 5 years (12 % of the total population). Study sites were selected based on where USAID Advancing Nutrition had programmes and where stunting prevalence was among the highest nationally at the start of the project in 2019. The intervention worked within the primary health care system in the Kyrgyz Republic, including Family Medicine Centers, General Medical Practice Centers and Feldsher-midwifery points. While the number and size of health facilities varied within the rayons, each rayon had several primary health care facilities that provide routine health and nutrition services to families.

### Intervention

The intervention centred on using the *RCEL Addendum* to counsel caregivers of children 0–23 months of age through two modalities: (1) individual counselling by health workers (i.e. family doctors and nurses) at primary health care facilities during routine well-child visits and (2) home visits by community-based activists (volunteers) who shared and discussed content using brochures (adapted from the *RCEL Addendum* counselling cards) with caregivers. Both cadres of counsellors received previous nutrition training (i.e. IYCF counselling).

**Fig. 1:**
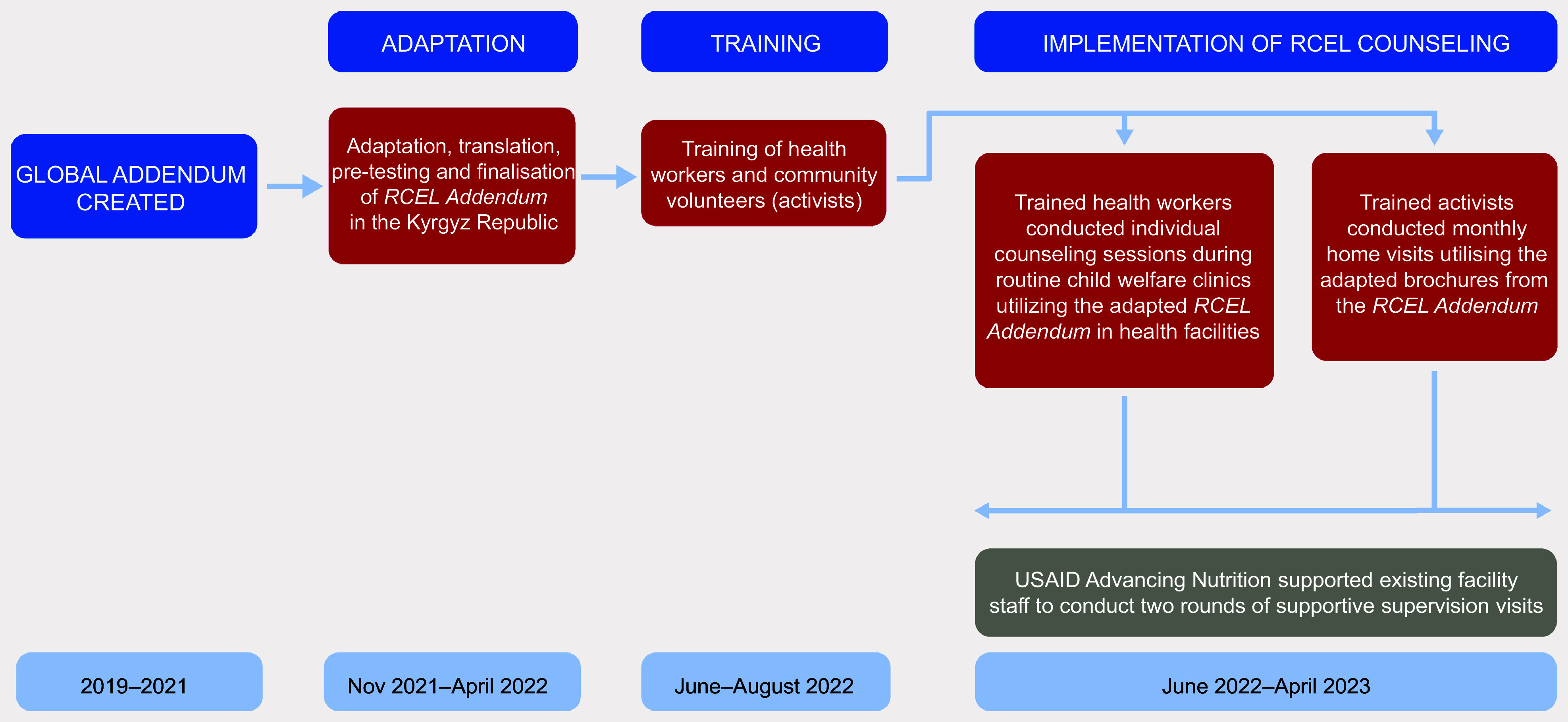
RCEL addendum development process and timeline in the Kyrgyz Republic

**Fig. 2:**
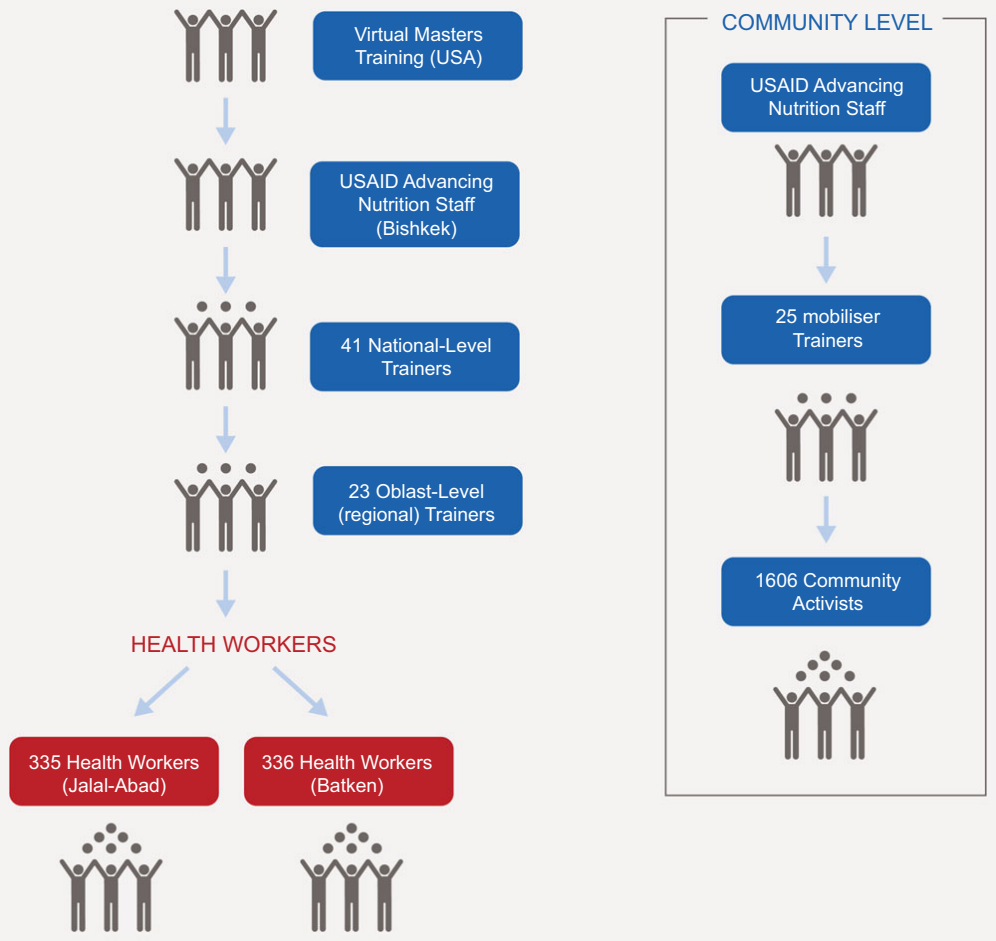
Cascade training approach in the Kyrgyz Republic

The intervention began with the adaptation of the global *RCEL Addendum* package to the Kyrgyz context, which included pre-testing the package, revising the content and images with a group of national experts who later served as master facilitators and translating it into Russian and Kyrgyz languages. Further details of the intervention development, adaptation and implementation are available elsewhere^(7)^ (Fig. 1). Following the adaptation, six national-level (master) facilitators trained twenty-three regional-level trainers, who then cascaded the training to 671 health workers in the Batken and Jalal-Abad oblasts during a 2-day training (Fig. 2). We also followed a cascade training approach at the community level. USAID Advancing Nutrition Kyrgyz master facilitators trained community mobilisers (staff hired by USAID Advancing Nutrition or the local civil society network to provide training, support and monitoring of community activists). The mobilisers then trained 1606 activists in both oblasts.

The community-level training used a modular training approach with 2-h sessions held 6 weeks apart (one session each on responsive care and feeding and early learning) and a refresher training held about 5 months following the initial modules. The trainings are modelled after the C-IYCF Counseling training and use proven participatory, adult learning approaches, focusing on experiential learning, mastery of one set of skills at a time and the practice of new skills. The training sought to improve counsellors’ knowledge of RCEL principles, improve individual and group counselling skills and highlight the importance of supportive supervision and mentorship to quality service provision.

Because the programme was integrated with existing health and nutrition services, there was no required number of RCEL contacts between health workers/activists and caregivers. Instead, the programme intended to provide RCEL counselling to caregivers during well-child visits at the health facility and during monthly activist home visits, of which four of the visits were intended to focus on RCEL, over a 10-month implementation period. To strengthen implementation quality, USAID Advancing Nutrition Kyrgyz Republic helped conduct two rounds of supportive supervision for RCEL counselling through the project’s existing IYCF counselling supportive supervision process^(7)^. Counsellor RCEL knowledge was tested before and after each training to assess uptake of knowledge, and we reassessed counsellors’ competencies to provide quality RCEL counselling during supportive supervision visits.

### Study design

We followed a cohort of participants over time and conducted a pre–post analysis to assess changes in RCEL practices, caregiver stress levels, IYCF practices and child supervision practices. Then, we examined the association between programme exposure and outcomes, controlling for likely confounders. The assessment included a household survey and observations to measure changes in RCEL practices. Baseline and endline data were collected by a local research firm from May to July 2022 and March to April 2023, respectively.

### Study population and sampling methods

The study included caregivers who had at least one child aged 0–23 months at baseline, were at least 18 years old and lived within the selected study rayons. Participants provided written informed consent to be included in the study.

Sample size was calculated based on a similar study in Bangladesh that estimated a maximum change in responsive caregiving practices of 15 percentage points from baseline to endline^(16)^. We sought to detect a 15-percentage point change in RCEL practices from pre- to post-intervention. We used a design effect of two to account for cluster sampling, 80 % power, 95 % confidence, assumed 10 % loss to follow up, and applied a continuity correction. With those parameters, this study needed to enrol 229 caregiver–child pairs which were randomly selected via a two-stage random cluster sampling strategy.

First, we created a list of eligible health facilities within the implementation area along with the number of eligible caregivers served by each facility and the cumulative population of the implementation area. We selected thirty clusters using a probability proportional to size approach. Then, we randomly selected eligible caregivers from each selected facility list (cluster) until the target sample size of at least eight caregiver–child dyads per cluster was achieved. The study team assigned unique identifiers for each caregiver–child dyad to ensure participants interviewed at baseline could be found and interviewed again at endline. We enrolled 237 caregivers of children 0–23 months of age at baseline and 220 of them completed the study and final assessment (7 % loss to follow up). Although the survey intended to interview and observe the same caregiver/child dyad at both baseline and endline, we interviewed nine new caregivers at endline, although the child remained constant throughout.

### Data collection

Members of the data collection firm received training on the purpose, methods, ethics and process of data collection from USAID Advancing Nutrition, and then conducted cascade training with their own staff (e.g. field team and interviewers). Select members of the data collection team (assessors) received additional training and practice on how to code caregiver responsiveness, the primary outcome of this study. Before starting the baseline data collection, assessors were required to reach an inter-rater reliability of their caregiver responsiveness coding score of 0·60 or higher^(17)^. Inter-rater reliability was determined based on a kappa coefficient to ensure that the assessors are consistent with the expert. Assessors were trained or re-trained before endline data collection and their inter-rater reliability on responsive care coding was assessed again. Eleven of the seventeen baseline assessors served again at endline. The six new assessors were paired with an experienced baseline assessor to ensure continuity and consistent quality. The survey instrument was translated into the Kyrgyz language and data were collected using a digital data collection tool, Kobo Collect Toolbox. Interviews were conducted in either Kyrgyz, Uzbek or Tajik languages based on the caregiver’s preference. The survey instrument was translated in Kyrgyz; other translations were performed by enumerators at the point of data collection.

### Variables measured

#### Primary outcomes

Primary outcomes were RCEL behaviours. Responsive care was measured using the *Responsive Care Observation Tool* developed by the Harvard T.H. Chan School of Public Health^(17,18)^. The tool uses a structured coding process during observation of 5-minute play sessions between a caregiver and child, which were recorded on video. To measure responsive care, two assessment team members reviewed each observation video and tallied the number of responsive behaviours (child-initiated responses), non-responsive behaviours (caregiver-initiated responses) and negative behaviours using a structured coding grid; all behaviours were also coded as verbal or non-verbal (average inter-rater reliability was 0·73 at baseline and 0·82 at endline). The percentage of each type of interaction (responsive, caregiver-initiated or negative) was calculated using the denominator of all the observed interactions during the 5-minute period. If the caregiver did not consent to being recorded, coding of the play session was done live (*n* 30). At endline, when the assessment team could not observe the interaction between the child and the caregiver (e.g. the child was sleeping or eating), twenty caregivers were instructed on how to make their own 5-minute videos, which they sent to the assessment team for coding. To create the opportunity for observation, we provided a novel item to the caregiver (e.g. a locally purchased toy or picture card) and asked that the caregiver interact normally with their child. We used *The Early Learning Tool* to assess whether children were engaged in stimulating activities. We summed the number of activities (maximum 14) conducted to create an indicator^(17)^.

#### Secondary outcomes

Secondary outcome measures included IYCF practices, parental stress and child supervision. IYCF indicators were measured using a 24-h dietary recall. We calculated minimum meal frequency, minimum dietary diversity and minimum acceptable diet for infants and children aged 6–23 months (at baseline and endline)^(19)^. Caregivers’ stress levels associated with parenting were assessed using the Parenting Stress Index, Fourth Edition, Short Form^(20)^. Stress was assessed on five measured indicators, each distinctively calculated as a sum score of different components of a thirty-five-item Likert scale questionnaire. The indicators included ‘parental distress’, ‘parent-child dysfunction’, ‘difficult child’, ‘total stress’ (each subscale has a potential score range from 12 to 60, with a total sum score possible range of 36–180) and status of high-level stress (based on scores in the 85th percentile or higher (yes/no), equivalent to a raw score of 110 or higher). Child supervision was assessed as being left alone or with another child under 10 years of age for 1 h or more in the week prior to the survey^(21)^.

#### Programme exposure

Programme exposure was measured at baseline and endline by asking caregivers how many times they visited a health facility and/or received a home visit by an activist in the past 6 months to discuss their child’s development. We summed the number of reported health facility and activist visits to obtain a total exposure number.

#### Socio-demographic indicators

We collected socio-demographic information using standard Multiple Indicator Cluster Survey (MICS) indicators, including age of child and caregiver, sex of child, caregiver education level, caregiver employment, literacy, nationality, relationship to the child and marital status, number of persons and children in the household, whether the father lived in the home and child’s screen exposure. We assessed primary caregiver functioning using the Washington Group Short Set on Functioning. Reporting ‘a lot of difficulty’ or ‘cannot do at all’ was used to categorise a caregiver as ‘has a disability’^(20)^. Screen exposure was measured using an adapted Seven-in-Seven Screen Exposure Questionnaire^(22)^, which included five items: daily screen time, viewing with parent(s), setting screen limits, screen exposure during meals and age of screen exposure onset. Each item was scored from 0 (low) to 3 (high) exposure. The endline questionnaire also included information on conflict exposure and displacement as a result of conflict in Batken in September 2022.

### Data analysis

All data were analysed using StataMP (v. 17, StataCorp)^(23)^. Descriptive summary statistics were calculated for the index child (age, sex) and caregiver characteristics (age, sex, level of education, literacy level, marital status). Point estimates and 95 % CI were calculated for all primary and secondary outcomes across the defined RCEL domains at both baseline and endline. We assessed the change in these indicators from baseline to endline using paired *t* tests or McNemar’s test for paired proportions depending on the outcome measure and applied a Bonferroni correction so that statistically significant change was indicated by *P*-values < 0·003.

To examine the association between programme participation and the outcomes, we first assessed the association of different demographic factors with these outcomes through bivariate analyses: caregiver–child interactions that were responsive, number of stimulating engagement activities by a caregiver with a child and caregivers reporting high parental stress. All factors that had a *P*-value less than 0·20 were then included as covariates (likely confounders) in multivariable logistic or linear regression models to assess the association of programme exposure with the prioritised outcomes, controlling for likely confounders. Additionally, we controlled for child age, caregiver education and the baseline measure of the outcome of interest in all models and applied survey weights. We controlled for each selected factor when inclusion did not prevent model convergence. To improve model performance, we used inverse probability of treatment weights for all modelled outcomes except for the number of negative interactions. We controlled for those factors in the regression equation for that model because inverse probability of treatment weight could not be calculated. The inverse probability of treatment weight were trimmed to exclude extreme outliers by removing 5 % of the total sample for all models that did not include conflict exposure covariates. Due to the small number affected, all models that included conflict exposure covariates retained their full samples.

To further assess if changes from baseline to endline were a result of children getting older, we conducted a sensitivity analysis to examine changes in all primary and secondary outcomes from baseline to endline for children who were 12–23 months at baseline (*n* 119) and those who were in that age group at endline (*n* 136). We used unpaired *t* tests and two sample tests of proportions to assess the differences between indicators at the two time points.

## Results

### Demographics and programme exposure

The mean age of the child was 12 months at baseline (Table 1). Caregivers were mostly Kyrgyz nationality (80 %). Overall, 93 % of caregivers had a secondary education or higher and over 99 % were literate. The mean number of persons living in the household was 6·5, with an average of 2·8 children under the age of 18. Less than 1 % of caregivers reported having a disability, 77 % of households reported the child’s father living in the home, 17 % of the children’s mothers had worked in the previous 12 months for money and 64 % of children had a grandparent involved in their care in the home. The majority of children (66 %) were reported to use screens with 5 % having a high level of screen exposure. At the endline, 45 % of participants from Batken oblast reported being forced from their home (either for a period of time or indefinitely) due to conflict in the region, and 13 % reported their access to health services had been disrupted. Regarding exposure to the intervention, at baseline, respondents reported discussing their child’s development with an activist on average less than one time (0·7) and a health worker at a health facility 3·5 times over a 6-month period. At endline, respondents reported discussing their child’s development with an activist on average less than one time (0·7) and a health worker at a health facility 2·5 times in the previous 6 months (Table 2).

**Table 1:** Participant demographics at baseline

Demographics	*n*	%
Region (Oblast)		
Jalal-Abad	121	50·7
Batken	99	49·3
Child’s sex		
Male	105	49·7
Female	115	50·3
Child’s age at baseline		
0–5 months	25	11·8
6–11 months	83	38·3
12–17 months	58	24·2
18–23 months	54	25·7
Caregiver’s relationship to child		
Biological mother	212	95·4
Grandparent	8	4·6
Caregiver marital status		
Single	6	3·5
In a partnership/living together	10	6·2
Married	204	90·3
Caregiver education		
Below secondary completed	15	7·8
Secondary completed	100	39·3
Higher than secondary completed	105	52·8
Caregiver literacy		
Not able to read and write	1	0·2
Able to read and write	219	99·8
Caregiver functioning		
Has a disability	2	0·3
No disability	218	99·7
Child’s father living in the home		
No	46	22·6
Yes	174	77·4
Child’s mother has worked in the previous 12 months for money		
Yes	37	17·2
No	183	82·8
A grandparent is involved in the care of the child in the home		
Yes	139	64·2
No	81	35·8
Nationality		
Kyrgyz	161	79·6
Uzbek	34	11·7
Tajik	22	7·4
Other	3	1·2
Screen time use		
Child uses screen device	150	65·5
High level of screen exposure	7	5·2
Exposure to conflict in Batken		
Family was displaced from their home because of the conflict in the region	38	44·8
Access to healthcare has been affected as a result of the conflict in the region	13	12·9
	Mean	se *
Household size		
Number of people in the household	6·5	0·2
Number of children under 18 years	2·8	0·1
Mother’s age at baseline	28·0	0·5

* se = Standard error.

**Table 2:** Participant reported programme exposure at baseline and endline

Programme exposure	Baseline		Endline	
	Mean	se *	Mean	se
Participated in activist meetings to discuss child’s development (mean number of meetings in the previous 6 months)	0·7·	0·2	0·7·	0·2
Visited a health facility to talk with a health worker about child’s development (mean number of visits in the previous 6 months)	3·5·	0·3	2·5·	0·3
Participated in activist meetings and/or visited a health facility to discuss child’s development (mean number of times in the previous 6 months)	4·2·	0·4	3·3·	0·4

* se = standard error.

### Changes in nurturing care practices

#### Responsive caregiving

**Fig. 3:**
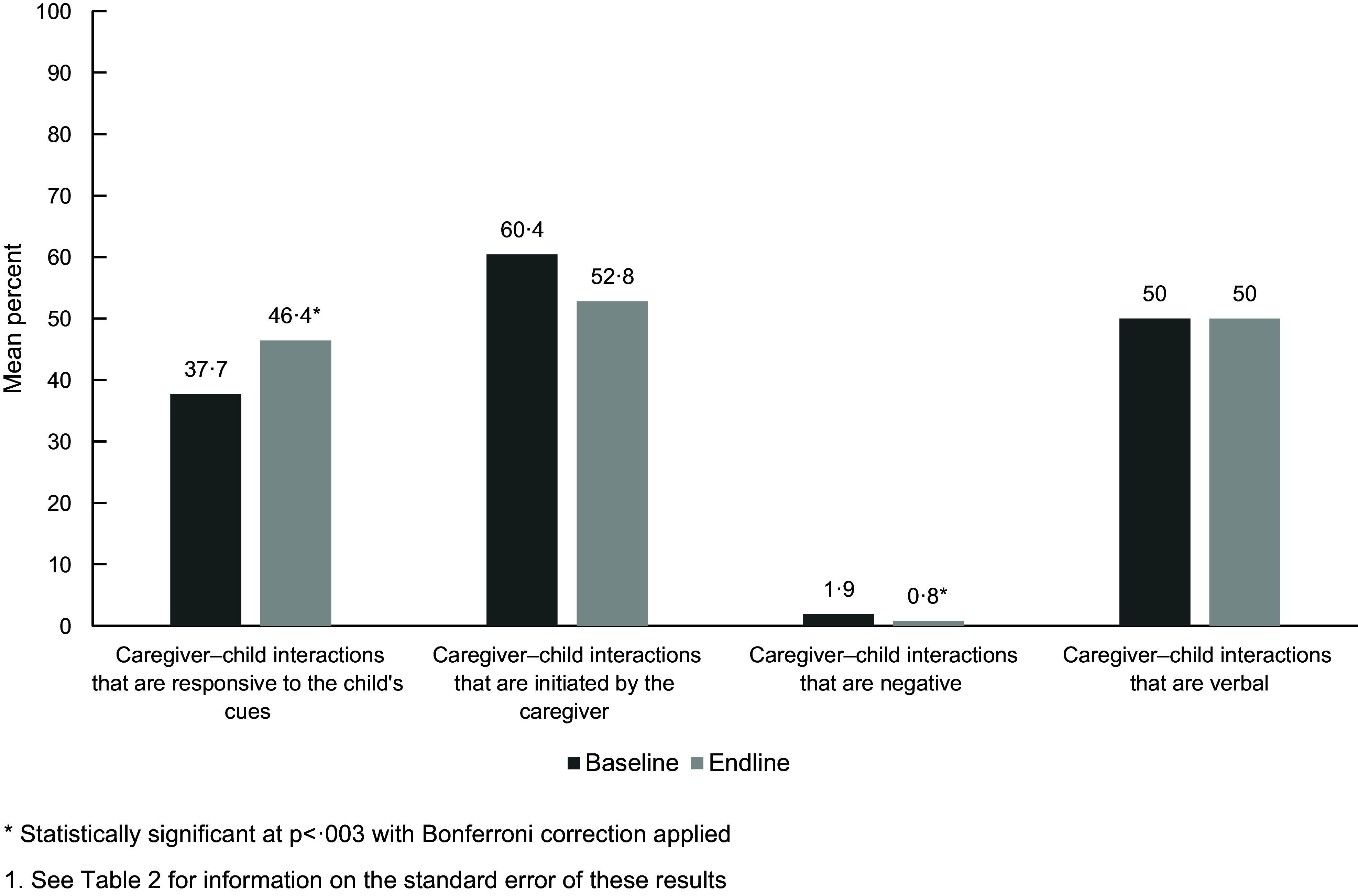
Changes in responsive caregiving practice

Results from the paired analysis indicate statistically significant changes in responsive caregiving practices assessed (*P* < 0·003) (Fig. 3; Table 3). Responsive caregiver–child interactions improved from 38 % at baseline to 46 % at endline (*P* = 0·002). There were slight but statistically significant reductions in caregiver–child interactions that were negative, reducing from 2 % to 1 %. Additionally, this slight decrease remained in the age sensitivity analysis (see online supplementary material, Supplemental Material 1). We found no difference in caregiver–child interactions that were verbal.

**Table 3:** Paired differences from baseline to endline

	Baseline % or mean*	se	Endline % or mean	se	Percentage point change or unit change	*P*-value†
Responsive care (*n* 214)‡						
Caregiver–child interactions that are responsive to the child’s cues	37·7·	2·5	46·4·	2·5	8·7	0·002†
Caregiver–child interactions that are initiated by the caregiver	60·4·	2·4	52·8·	2·4	−7·6	0·007
Caregiver–child interactions that are negative	1·9·	0·4	0·8·	0·2	−1·1	< 0·001†
Caregiver–child interactions that are verbal	50·0·	1·2	49·5·	1·2	−0·5	0·665
Early learning (*n* 220)§						
Number of stimulating engagement activities by a caregiver with a child from 0 to 23 months with objects (e.g. playthings) and/or people (adults and peers)§	8·0·	0·2	9·6·	0·2	1·6	< 0·001†
Parenting stress (*n* 220)||						
PD sub-scale score	29·6·	0·5	27·3·	0·7	−2·3	0·012
P-CDI sub-scale score	33·2·	0·6	29·6·	0·4	−3·6	< 0·001†
DC sub-scale score	26·8·	0·6	32·1·	0·4	5·2	< 0·001†
Total stress score	89·6·	1·5	88·9·	1·3	−0·7	0·66
Caregivers reporting high parental stress	7·7		6·1		−1·6	0·741
Exposure to inadequate supervision (*n* 220)§						
Left alone in the past week	19·7		15·7		−4·0	0·295
Left under the supervision of another child younger than 10 years of age in the past week	19·1		23·9		4·8	0·185
Left with inadequate supervision in the past week	29·3		30·4		1·2	0·734
Infant and young child feeding (*n* 113)¶						
Children 6–23 months who are achieving MDD	64·2		80·5		16·3	0·008
Children 6–23 months who are achieving MMF	38·7		79·3		40·6	< 0·001†
Children 6–23 months who are achieving MAD	32·8		62·2		29·5	< 0·001†

PD, parental distress; P-CDI, parent–child dysfunction; DC, difficult child; MDD, minimum dietary diversity; MMF, minimum meal frequency; MAD, minimum acceptable diet.

* Mean and se reported for paired *t* tests; percentage reported for McNemar’s tests.

† Statistically significant at *P* < 0·003 with Bonferroni correction applied.

‡ Assessed using the Responsive Care Observation Tool.

§ Assessed using The Early Learning Tool out of a maximum possible score of 14; see online supplementary material, Supplemental Material 3 for the full list of stimulating engagement activities included in the tool.

|| Assessed using the Parenting Stress Index, Fourth Edition, Short Form. Subscale scores range from 12 to 60 with a total sum score range from 36 to 180. High stress is defined as a raw total stress score of 110 or higher, which is equivalent to the 85 percentile.

¶ Assessed using a 24-h dietary recall.

#### Early learning

We saw a statistically significant improvement in the mean number of engagement opportunities in the home from 8·1 at baseline to 9·6 at endline (*P* < 0·001) (Table 3). This was no longer significant in the age sensitivity analysis (see online supplementary material, Supplemental Material 1).

#### Safety and security

Data on child supervision show a small, but non-statistically significant increase in inadequate supervision from baseline to endline.

#### Infant and young child feeding

We found statistically significant improvement in two of the three complementary feeding indicators: minimum dietary diversity improved from 64 % to 81 % but that was not a statistically significant increase (*P* = 0·008). Minimum acceptable diet improved from 33 % to 62 % (*P* < 0·001) and minimum meal frequency improved from 39 % to 79 % (*P* < 0·001) from baseline to endline. Minimum acceptable diet and minimum meal frequency changes remained statistically significant in the age sensitivity analysis.

#### Parental stress

Parental distress and parent–child dysfunction showed significant reductions from baseline to endline (*P* < 0·003), but a five-point increase within the difficult child domain (*P* < 0·001). The decrease in parent–child dysfunction and increase in the difficult child domain remained statistically significant in the age sensitivity analysis (see online supplementary material, Supplemental Material 1).

### Associations between programme exposure and outcomes

Factors with *P* < 0·20 were included in the multivariable analysis as likely confounders: caregiver nationality, oblast, father living in the home, child’s screen exposure, mother’s age, number of household members, displacement of the family from their home because of conflict, and whether family accommodated/supported others that were affected by conflict.

Increased programme exposure was associated with a decrease of 23 % in reported parental stress (RR: 0·77, 95 % CI: (0·69, 0·96)) and an increase in inadequate supervision in the past week (RR: 1·04, 95 % CI: (1·01, 1·08)) (Table 4). Programme exposure was not associated with other outcomes of interest.

**Table 4:** Multivariable regression analysis of caregiver’s program exposure and prioritised outcomes of interest

	Caregivers reporting high parental stress^↑††^ (*n* 209)		Children left with inadequate supervision in the past week† (*n* 143)		Number of caregiver-child interactions that were responsive¶ (*n* 93)		Number of stimulating engagement activities by a caregiver§ (*n* 209)	
	RR	95 % CI	RR	95 % CI	RD	95 % CI	RD	95 % CI
Program exposure*	0·90	0·77, 1·05	1·08	0·99, 1·18	−0·56	−2·01, 0·88	−0·04	−0·23, 0·15

	Caregivers reporting high parental stress‡‡ (*n* 99)		Children left with inadequate supervision in the past week|| (*n* 71)		Number of caregiver–child interactions that were responsive‡ (*n* 204)		Number of stimulating engagement activities by a caregiver** (*n* 99)	
	RR	95 % CI	RR	95 % CI	RD	95 % CI	RD	95 % CI
Program exposure*	0·77	0·69, 0·86	1·04	1·01, 1·08	−0·33	−1·16, 0·49	−0·05	−0·12, 0·01

¶ Controlled for baseline measure, child age, caregiver’s education, oblast, displacement of the family from their home because of conflict and whether the family accommodated/supported others that were affected by conflict.

§ Controlled for baseline measure, child age, caregiver’s education, oblast and mother’s age.

* Program exposure was defined as the number of times a caregiver visited a health facility or participated in activist group meetings to discuss their child’s development.

|| Controlled for baseline measure, child age, caregiver’s education, oblast, father living in the home, screen exposure, mother’s age, number of household members and displacement of the family from their home because of conflict.

‡ Controlled for baseline measure, child age, caregiver’s education, oblast, father living in the home, screen exposure, mother’s age and number of household members.

** Controlled for baseline measure, child age, caregiver’s education, oblast, mothers age and whether the family accommodated/supported others that were affected by conflict.

†† Controlled for baseline measure, child age, caregiver’s education, and nationality.

‡‡ Controlled for baseline measure, child age, caregiver’s education, nationality, and displacement of the family from their home because of conflict.

## Discussion

We found improved caregiving practices and increased early learning opportunities following the delivery of RCEL counselling using the *RCEL Addendum*, through existing facility- and community-based platforms providing nutrition services in the participating areas of the Kyrgyz Republic. We observed significant increases in RCEL practices from pre- to post-intervention and decreases in parental stress. The paired results also showed statistically significant improvements in IYCF practices (i.e. minimum meal frequency and minimum acceptable diet increased by 41 and 30 percentage points, respectively, between baseline and endline), suggesting that caregivers improved both RCEL and IYCF practices and that integration did not negatively affect the delivery of nutrition services through these same delivery points.

However, the age sensitivity analysis indicated that some of the statistically significant results from the paired analysis may have been due to children’s maturation with age. As the children aged, they were more likely to be engaged. This increased level of engagement is not surprising, as older children can typically express what they want more clearly, and caregivers may perceive it to be easier to interact with and play with an older child given their increased range of abilities (i.e. walking and talking) and demand for attention. These findings highlight the need in the Kyrgyz Republic to continue to discuss how caregivers can engage with very young children (less than a year old) and continue to build caregivers’ abilities to identify, understand and respond to the youngest children’s cues.

When we controlled for likely confounders, we found that programme exposure was associated with reduced caregiver stress and a slight increase in inadequate supervision but not specifically with improved RCEL practices. These findings could be due to the use of a limited exposure indicator which asked whether caregivers discussed ‘child development’ in general with providers. Caregivers may not have distinguished between RCEL and nutrition counselling. USAID Advancing Nutrition had supported nutrition programming (i.e. IYCF counselling and growth monitoring and promotion – which assesses a child’s physical development) over the past 2 years, using the same cadre of health workers and activists, which may have caused confusion over what constituted counselling on child development. Furthermore, since the nutrition programming was ending at the time of the RCEL baseline, this confusion could help explain why exposure numbers were higher at baseline than endline, when the nutrition programming had ended. Furthermore, implementation of the intervention through existing government services did not allow us to ensure higher exposure levels. On average, during 6 months prior to endline data collection, caregivers discussed their child’s development 2·5 times with health workers. This exposure is low compared to previous effectiveness studies, where contact frequency was typically weekly or fortnightly^(24–26)^. The Kyrgyz Republic Ministry of Health recommends that children 0–12 months receive well-child visits every 1–2 months, children 1–2 years every 3 months and children 2–3 years every 6 months^(27)^. If current recommendations were followed, we could have seen higher levels of exposure, although it would depend on the age of the child. Lower endline exposure could also be due to a portion of the children ageing out of the more frequent well-child visit schedule. At endline, 63 % of children in the study were between 1 and 2 years of age and 36 % percent were between 2 and 3 years of age; requiring less frequent visits as they aged.

In the future, efforts to increase the frequency and intensity of the intervention could have a greater likelihood of promoting positive changes in practices. Such efforts could include greater use of activists and other community networks to promote and support RCEL practices^(28,29)^. In addition, global research highlights the importance of quality pre-service and in-service training and supportive supervision to the provision of quality counselling^(30–32)^. Programme data show improvements in health workers RCEL counselling skills between two rounds of supportive supervision during the 10-month implementation period, indicating the importance of on-site supervision and practice with the new RCEL content^(7)^. Therefore, continued investment in both training and supportive supervision to ensure quality counselling, and appropriate intensity of the intervention, is suggested. Lastly, complementary approaches using videos, social and mass media (as of 2021 78 % of the population in the Kyrgyz Republic uses the internet^(33)^), and traditional media (e.g. posters) may help bring attention to and raise demand for RCEL counselling. These activities could also help to reinforce and support RCEL counselling content^(34)^.

An important strength of this study was delivery of the intervention entirely through the existing government system and associated staff. Given the importance of RCEL to ECD outcomes, evidence of feasible, acceptable and effective opportunities to build upon existing structures to give caregivers and children more holistic care is critical. While we did provide additional financial support to train and supervise health workers, the supportive supervision process was mostly built upon existing platforms and was integrated with IYCF supervision processes and checklists. Programme supervision data indicate that these supportive supervision efforts were critical in strengthening the RCEL counselling skills of the health workers^(7)^.

This study had several limitations. First, we used a pre–post cohort design without a comparison group or random assignments, among participating families. Therefore, we cannot determine whether the observed changes are directly attributable to the *RCEL Addendum* intervention or if similar results would be observed with a random population sample. Due to the lack of a comparison group, it is possible that observed changes could have resulted from ambient exposure to the messaging and not specifically due to the role of the activists or health workers themselves. Furthermore, while the use of a cohort study design helped us examine changes in practices within individual caregiver–child pairs, it meant that children at endline were approximately 10 months older than at baseline, and some of the results observed could have been due to the children’s ageing. While we did conduct a sensitivity analysis to assess the impacts of age on our outcomes, we had limited statistical power to do so, affecting our understanding of those results. In addition, there is the potential for the Hawthorne effect, whereby study participants may change their behaviour because they are aware that they are being observed, thus potentially influencing the results. Similarly, some of our findings may have been influenced by reporting bias, with participants reporting practices that they knew were optimal, or simply from parents hearing about RCEL and adopting the practices they see friends employing. Another limitation was our inability to measure the baseline and endline at the exact same time of year, with seasonality possibly influencing some of our results. In addition, the use of a basic exposure indicator examining only the frequency of contacts with counsellors, as opposed to a more robust measure that asked caregivers to report back what they discussed in a specific contact or an observational measure of the content and quality of contacts, limited our ability to fully capture and understand programme exposure. Lastly, due to programmatic constraints, we could not enforce greater intensity through the existing government system and measured change after only 10 months of implementation. It is possible that greater improvements would be seen with a longer duration of more intense programming (e.g. 18–24 months instead of 10 months), ensuring caregivers of all children involved in the study received counselling throughout the first 2 years of their child’s life at a consistent frequency (i.e. once every 1–2 months for children 0–12 months of age and every 3 months for children 1–2 years of age).

### Conclusion

Our findings show increased responsive caregiving practices and early learning opportunities among caregivers who received RCEL counselling through existing facility and community-based health and nutrition platforms in the Kyrgyz Republic. These changes in caregiver RCEL practices are important, as improvements are associated with positive infant cognitive, language, motor and development outcomes^(35)^. Furthermore, this integration did not appear to disrupt nutrition service delivery or have a negative impact on complementary feeding outcomes, but rather suggest synergistic benefits. However, potential benefits may be amplified with increased duration and intensification of the intervention and support with complementary activities. Given the importance of providing holistic care to support optimal ECD, these findings provide new evidence on how to strengthen the delivery of nurturing care services in the Kyrgyz Republic. Future implementation efforts should create an enabling environment for optimal RCEL practices by using multiple touchpoints to reach caregivers. Additionally, future programmes should ensure a focus on high-quality implementation, with training and supportive supervision processes to support high-quality counselling. Future research could focus on how to build caregivers’ abilities to respond and engage with the youngest children (less than a year old) and more directly assess the effect of the *RCEL Addendum* counselling through measurement of ECD outcomes.

## Supporting information

Oot et al. supplementary material Oot et al. supplementary material

## Supplementary material

For supplementary material accompanying this paper visit https://doi.org/10.1017/S1368980024001642
