# Supplementary material for: Promoting responsive care and early learning practices among caregivers of children 0–23 months in the Kyrgyz Republic: findings from integrating a counselling intervention with nutrition services
Source: Public Health Nutr. 2024 Oct 8;27(1):e202. doi: 10.1017/S1368980024001642 (PMC11604327; doi:10.1017/S1368980024001642)
Supplement: Oot et al. supplementary material [file S1368980024001642sup001.docx]

**SUPPLEMENTARY MATERIALS**

**Supplementary Material 1: Unpaired Baseline and Endline Differences for Children 12–23 Months Old at Baseline or Endline**

| Indicator | Baseline (unweighted n=119) | | Endline (unweighted n=136) | | Change Baseline to Endline |
| --- | --- | --- | --- | --- | --- |
|  | % or Mean (SE)* | | % or Mean (SE)* | | Percentage point change or unit change |
| Caregiver-child interactions that are responsive to the child’s cues | 41.8 | (2.4) | 43.01 | (2.4) | 1.21 |
| Caregiver-child interactions that are initiated by the caregiver | 56.39 | (2.3) | 56.13 | (2.3) | -0.26 |
| Caregiver-child interactions that are negative | 1.82 | (0.4) | 0.87 | (0.3) | -0.95† |
| Caregiver-child interactions that are verbal | 51.64 | (1.8) | 47.97 | (1.6) | -3.67 |
| Number of stimulating engagement activities by a caregiver with a child from 0–23 months with objects (e.g., playthings) and/or people (adults and peers)* [Scores can range from 0 to 14] | 9.52 | (0.2) | 10.2 | (0.6) | 0.68 |
| PD Sub Scale Score [Scores can range from 12 to 60] | 28.81 | (0.6) | 27.54 | (1.0) | -1.27 |
| P-CDI Sub-Scale Score [Scores can range from 12 to 60] | 33.64 | (0.5) | 29.29 | (0.5) | -4.35† |
| DC Sub-Scale Score [Scores can range from 12 to 60] | 27.06 | (0.6) | 31.67 | (0.6) | 4.61† |
| Total Stress Score [Scores can range from 36 to 180] | 89.51 | (1.46) | 88.49 | (1.8) | -1.02 |
| Caregivers reporting high parental stress [Total stress score of 110 or higher] | 9.3 |  | 8.15 |  | -1.15 |
| Left alone in the past week | 24.46 |  | 18.98 |  | -5.48 |
| Left under the supervision of another child younger than 10 years of age in the past week | 21.12 |  | 23.88 |  | 2.76 |
| Left with inadequate supervision in the past week | 32.13 |  | 31.85 |  | -0.28 |
| Children 12–23 months who are achieving MDD | 81.98 |  | 79.37 |  | -2.61 |
| Children 12–23 months who are achieving MMF | 50.46 |  | 78.94 |  | 28.48† |
| Children 12–23 months who are achieving MAD | 38.06 |  | 60.73 |  | 22.67† |

*Mean and standard error reported for paired t-tests; Percentage reported for McNemar’s tests.

† Significant at p<0.05

**Supplementary Material 2: Bivariate Association of Prioritized Behaviors with Prioritized Outcomes of Interest**

| Factors | Caregivers reporting high parental stress | | Children 6–23 months who are achieving minimum acceptable diet | | Number of caregiver-child interactions that were responsive | Number of stimulating engagement activities by a caregiver | Number of caregiver-child interactions that are negative |
| --- | --- | --- | --- | --- | --- | --- | --- |
|  | % or mean | | % or mean | | mean | mean | mean |
|  | Yes | No | Yes | No |  |  |  |
| Caregiver Education |  |  |  |  |  |  |  |
| Below secondary | 18.6 | 5.4† | 3.9 | 10.5 | 40.3 | 9.9 | 1.5 |
| Secondary completed | 34.1 | 40.0† | 31.6 | 41.1 | 47.8 | 9.5 | 0.8 |
| Higher than secondary completed | 47.3 | 54.6† | 64.6 | 48.4 | 46.1 | 9.6 | 0.7 |
| Child’s Father Living in the Home | | | | | | | |
| Yes | 71.2 | 79.4 | 80.3 | 84.0 | 46.1 | 9.6 | 0.8 |
| No | 28.8 | 20.6 | 19.8 | 16.0 | 47.4 | 9.5 | 0.8 |
| Child Sex |  |  |  |  |  |  |  |
| Male | 35.9 | 51.3 | 42.4 | 48.1 | 47.5 | 9.4 | 0.9 |
| Female | 64.1 | 48.7 | 57.6 | 51.9 | 5.3 | 9.7 | 0.8 |
| Screen Exposure |  |  |  |  |  |  |  |
| Low | 100.0 | 94.5 | 100.0 | 90.8† | 47.2 | 9.5 | 0.9* |
| High | 0.0 | 5.5 | 0.0 | 9.2† | 53.5 | 11.1 | 0.1* |
| Nationality |  |  |  |  |  |  |  |
| Kyrgyz | 68.6 | 82.9† | 86.0 | 78.0 | 46.8 | 9.5 | 0.7 |
| Uzbek | 27.7 | 10.2† | 8.7 | 12.1 | 40.9 | 10.0 | 2.2 |
| Tajik | 3.6 | 5.6† | 5.3 | 6.5 | 51.5 | 9.6 | 0.0 |
| Other | 0.0 | 1.3† | 0.0 | 3.4 | 44.0 | 7.8 | 0.6 |
| Family Forced from Home Because of Conflict | | | | | | | |
| Yes | 81.6 | 43.0** | 61.4 | 45.7† | 45.3* | 10.0 | 0.1 |
| No | 18.4 | 57.0** | 38.6 | 54.3† | 60.0* | 9.7 | 0.2 |
| Family Accommodated/Supported Others Affect by Conflict | | | | | | | |
| Yes | 46.9 | 45.1 | 52.3 | 40.9 | 56.8† | 10.9* | 0.2 |
| No | 53.1 | 54.9 | 47.7 | 59.1 | 50.7† | 8.9* | 0.1 |
| Oblast |  |  |  |  |  |  |  |
| Jalal-Abad | 66.7 | 56.2 | 59.2 | 50.3 | 41.0* | 9.4† | 1.3* |
| Batken | 33.3 | 43.8 | 40.8 | 49.8 | 53.4* | 9.8† | 0.1* |
|  |  |  |  |  | β (95% CI) | β (95% CI) | β (95% CI) |
| Child’s Age | 10.6 | 12.2 | 8.7 | 8.3 | 0.82 (0.35, 1.29) | 0.06 (-0.02, 0.14)† | -0.02 (-0.05, 0.01) |
| Mother’s age | 28.3 | 28.3 | 28.9 | 27.5* | -0.10 (-0.45, 0.24) | 0.11 (0.03, 0.20) | -0.02 (-0.08, 0.04) |
| Number of HH Members | 6.2 | 6.5 | 6.4 | 6.4 | -0.30 (-1.24, 0.64) | 0.02 (-0.15, 0.19) | -0.04 (-0.12, 0.05) |
| Mean Number of Times a Caregiver Participated in an Activist Meeting or Visited a Health Facility to Discuss their child’s development | 3.3 | 3.3 | 3.4 | 3.3 | -0.33 (-1.52, 0.85) | 0.09 (-0.07, 0.25) | -0.01 (-0.09, 0.08) |

* Statistically significant at p<0.05

† Statistically significant at p<0.20 for inclusion in multivariate regressions

**Supplementary Material 3: Stimulating engagement activities included in the Early Learning Tool**

| - Things for moving around (balls, wheels, push & pull) |
| --- |
| - Things for role-playing, pretending (e.g., dolls, household items, plane, cars) |
| - Things to manipulate: to fill, stack, construct, build (blocks, sticks, stones) |
| - Things that produce sound (e.g., drum) |
| - Child has picture book (not textbook, can be collection of pictures) |
| - Things with different shapes and colors, or for drawing shapes, colors |
| - Is there a designated and accessible place for child’s playthings? |
| - Did you read or look at pictures in a book, calendar or magazine with the child in the last 24hours? |
| - In the last 24 hours, did you take your child out to visit friends, family or to shop? |
| - In the last 24 hours, did you tell stories or rhymes to the child? |
| - In the last 24 hours, did you sing songs or lullabies with the child? |
| - In the last 24 hours, did your child play any structured games with people, like circle games, clapping, singing or ones with objects? |
| - When you are busy with housework, in the last 24 hours, did you talk with your child? |
| - In the past week, did you give your child a new plaything? (What?) |
